# Selective Electrochemical Conversion of CO_2_ into Methane on Ag‐Decorated Copper Microsphere

**DOI:** 10.1002/open.202400173

**Published:** 2024-10-24

**Authors:** Rabin Dahal, Rohit Srivastava, Bishnu Prasad Bastakoti

**Affiliations:** ^1^ Department of Chemistry North Carolina A & T State University 1601 E Market St Greensboro, NC 27411 USA; ^2^ Catalysis & Hydrogen Research Lab Department of Petroleum Engineering School of Energy Technology Pandit Deendayal Energy University Gandhinagar, Gujarat India

**Keywords:** CO_2_ reduction, Bimetallic catalyst, Methane formation, Faradaic efficiency

## Abstract

We synthesized the silver‐decorated copper microsphere via the hydrothermal method followed by photoreduction of silver ions. Sub 100 nm Ag nanoparticles anchored on the surface of Cu microspheres enhance the electrochemical performance and the selectivity of the CO_2_ reduction into CH_4_. Incorporating Ag nanoparticles onto Cu lowers the charge transfer resistance, enhancing the catalyst's conductivity and active site and increasing the rate of CO_2_ reduction. The faradaic efficiency of silver nanoparticles decorated copper microsphere for methane was 70.94 %, almost twice that of a copper microsphere (44 %). The electrochemical performance showed higher catalytic properties, stability, and faradaic efficiency of silver‐decorated copper microspheres.

## Graphical abstract

The photoreduction of silver ions on the preformed Cu microspheres leads to the formation of Ag‐decorated Cu microspheres to boost the electrochemical conversion of CO_2_ into Methane. The electrochemical performance of Ag‐decorated Cu microspheres showed higher catalytic properties, stability, and faradaic efficiency than that of undecorated Cu microspheres.

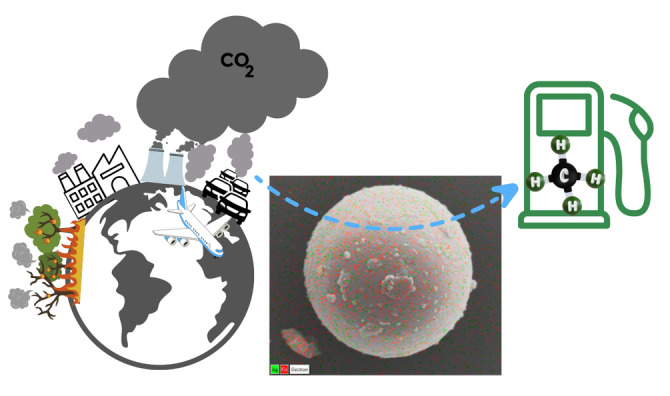

## 1 Introduction

The leading issue of climate change due to excessive CO_2_ emission by massive utilization of fossil fuels is the biggest threat to the world. Similarly, the excessive use of fossil fuels also leads to an energy crisis in the future. Both issues can be resolved by utilizing CO_2_ to generate renewable energy sources.[^1^, ^2^, ^3^, ^4^] So, electrochemical reduction of CO_2_ is getting much more attention due to its low cost, mild reaction conditions, and higher capability of forming value‐added chemicals/fuels.^[5]^ For this, much effort has been given into designing an effective CO_2_ reduction catalyst with improved activity and selectivity.^[6]^ Multiple products (CO, CH_3_OH, C_2_H_4_, CH_4_, HCOOH, C_2_H_5_OH) are formed during electrochemical CO_2_ reduction reaction (CO_2_RR).[^7^, ^8^] Therefore, a perfectly designed catalyst is needed to achieve a specific level of selectivity with enhanced electrochemical activity.^[9]^ Different approaches, including nano‐structuring, reaction environment adjustment, and active site control through catalyst composition and structure, have demonstrated notable advancements toward selective and effective CO_2_RR conversion.^[10]^ Among the metallic catalysts, Cu is a promising catalyst used for electrochemical CO_2_ reduction due to its selectivity towards hydrocarbon and the formation of multiple products like C1 and C2 (C−C coupling) depending on experimental conditions.^[11]^ However, its stability, selectivity, and optimum catalytic activity are major concerns that need to be modified. Multiple research studies have been carried out to enhance the physicochemical properties by maneuvering its chemical state, shape, size, grain boundaries, and surface morphology for the effective improvement of catalytic behavior as well as the selectivity of the product.^[12]^

Bimetallic catalysts perform better than individual metals in CO_2_RR due to electronic structure, more significant synergistic effect, improved CO_2_ adsorption, greater active site, and effective charge transfer.[^13^, ^14^] So, incorporating a second metal into Cu is gaining interest in improving the electrochemical properties. Xu and coworkers used Cu−Ag alloy to enhance the selectivity of the C_2_ liquid product by suppressing the O−C−O intermediate forming C1 product.^[15]^ Kaneco et al. used Cu electrodes in the presence of different lithium salt/methanol‐based electrolytes at low temperatures (−30 °C) for electrochemical CO_2_ reduction.^[16]^ Ni−Cu alloys were used by Song et al. to convert C_1_ products into major C_2_ products with two‐fold increments.^[17]^ Thiet et al. synthesized CuZn‐MOF for high selective reduction of CO_2_ into CO.^[18]^ Dendritic Ag−Cu prepared by electrodeposition was used for the selective reduction of CO_2_ into CO by Choi et al..^[19]^ Formate was formed on CuBi‐MOF in the electrochemical reduction of CO_2_.^[20]^

Here, we synthesized a silver‐decorated copper microsphere where silver significantly increases CO binding affinity and enhances the electrochemical properties that greatly affect the product's selectivity. Incorporating silver over copper provides a synergetic effect and increases the multiple active sites for the adsorption of *CO to increase methane production. Methane can be used directly as a fuel, a clean and renewable energy source. So, it can be used as an alternative source that can replace the existing use of fossil fuels and somewhat compensate for the energy crisis.

## Experimental Section

### Catalyst Synthesis

The chemicals used were of analytical grade and were used without further purification. Copper nitrate trihydrate [Cu(NO_3_)_2_.3H_2_O] was purchased from Acro's Organic, Poland. The sodium salt of ascorbic acid (99 %) was purchased from Acro's Organics, Belgium; silver nitrate was purchased from Fisher Scientific, Belgium, and Ethanol [C_2_H_5_OH (95 % pure)] was purchased from VWR Chemicals, Canada. The catalyst was synthesized using a hydrothermal method followed by photoreduction. 2 gm of copper nitrate was weighed and dissolved in 40 mL of DI water. Then 2 gm of sodium salt of ascorbic acid was added into it. The mixture was transferred into a 100 mL Teflon flask. The flask was inserted into the autoclave and kept in a vacuum oven at 120 °C for 4 hours, and it was cooled naturally. The copper microsphere obtained was washed three times with water and ethanol via centrifugation. The sample was dried in a vacuum oven. Again, 2 gm of synthesized copper microsphere was weighed and added to 100 mL of ethanol (95 %). The solution was stirred for an hour, followed by the addition of 150 mg of silver nitrate into the solution. The solution was irradiated with a UV lamp for 4 hours in a closed condition. The solution was washed with water (3 times) and finally with ethanol via centrifuging. The prepared catalyst was dried in a vacuum oven at room temperature. Finally, the prepared catalyst was ground and subjected to different characterization and electrochemical performance.

### Material Characterization

Powder x‐ray diffraction (XRD) patterns were recorded on a diffractometer in Rigaku Miniflex 600 (2: 30–80, step: 0.02, and continuous: 2°/min). The morphology of samples was obtained by field emission scanning electron microscopy (FESEM, JEOL, JSM‐IT800). Transmission electron microscope (TEM) and high‐resolution (HRTEM) images of the prepared samples were obtained from JEOL JEM‐2100 plus at 120 kV. The X‐ray photoelectron spectroscopy (XPS) was carried out on Thermo Scientific ESCALAB^TM^ XI (200 eV and Al Kα). Gas chromatography (GC) (SRI 8610 C) analyzed the gaseous products. The electrochemical measurements were performed on CH instruments.

### Electrochemical Measurements

The electrochemical measurements were performed in an H‐type cell with a three‐electrode system. The H‐type cell consists of platinum (as a counter electrode), Ag/AgCl electrode (as a reference electrode stored in KCl solution), and sample (as a working electrode) in 0.5 M KHCO_3_ solution. The electrode was prepared by mixing the 4 mg sample and 50 μL Nafion in 400 μL ethanol. The mixture was sonicated for 60 min. FTO glass (MSE 2.2 mm, 12–15 ohm/sq, TEC 15 coated glass substrate) was used as a substrate to prepare the electrode after washing with DI water and ethanol. The highly dispersed mixture was deposited on the 1 cm ×1 cm FTO glass and left to dry for 12 hours in an oven at 60 °C. The cyclic voltammetry (CV) of the prepared materials was measured with a scan rate between 60 mV/s to 100 mV/s. Similarly, the conductivity of the electrode was studied by electrochemical impedance spectroscopy (EIS) between 0.1 Hz to 100,000 Hz. Likewise, the linear sweep voltammetry (LSV) was studied between −0.25 to −1 V vs the reversible hydrogen electrode (RHE) at the scan rate of 10 mV/s. The following equation was used for the calculation of RHE:

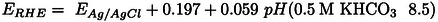




where E_Ag/AgCl_ represents the potential against the reference electrode, and 0.197 denotes the standard potential of Ag/AgCl at 25 °C.^[21]^

A 50 mL H‐type electrochemical cell was used. Nafion membranes were used after further treatment with 0.1 M H_2_SO_4_ acid and DI water to separate the two compartments (anodic and cathodic). For the saturation of electrolytes, 99.99 % pure CO_2_ gas was bubbled in a cathodic cell compartment for 60 min with 15 sccm with the help of a mass flow controller (MC‐100SCCM−D, Alicat Scientific). The outlet of the cathodic cell compartment was connected to GC. GC was calibrated with a standard gas mixture (ARC3). The gaseous products obtained during the reaction were detected by a flame ionization detector. The current‐time (it) measurements were performed at a fixed potential of −0.50 V Vs RHE. Gasses from the cell were injected into GC for 400 sec, and electrocatalytic CO_2_RR was evaluated. The stability of the microsphere was conducted over 12 hours.

## 2 Results and Discussion

**Figure 1:**
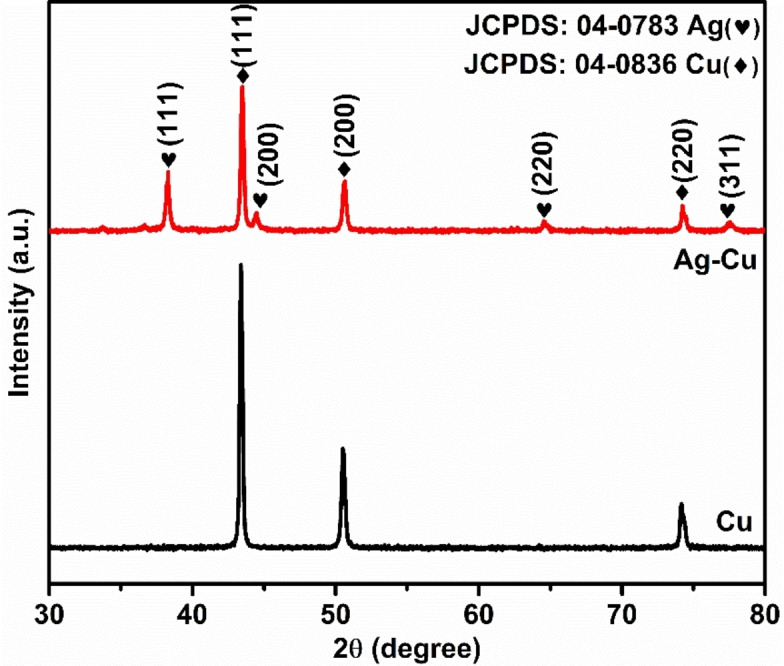
XRD spectrum of the copper and silver‐decorated copper microsphere.

Figure 1 revealed the XRD patterns of copper microspheres and silver‐decorated copper microspheres. As observed, the peaks at 43.2°, 50.1° and 74.1° are (111), (200), and (220), respectively for the face‐centered cubic structure of metallic copper with strong (111) orientation plane (JCPDS: 04–0836) and the peaks at 38.1, 44.2°, 64.4° and 77.4° assign to (100), (200), (220), and (311) plane respectively and correspond to the face‐centered cubic structure of silver peaks (JCPDS: 04–0783).[^9^, ^22^] Those peaks indicate the polycrystalline nature of Cu and Ag. Shifting of the peak was not seen, which suggests no alteration in the lattice plane. The individual peaks of Cu and Ag show the formation of a bimetallic phase instead of an alloy phase.^[23]^

**Figure 2:**
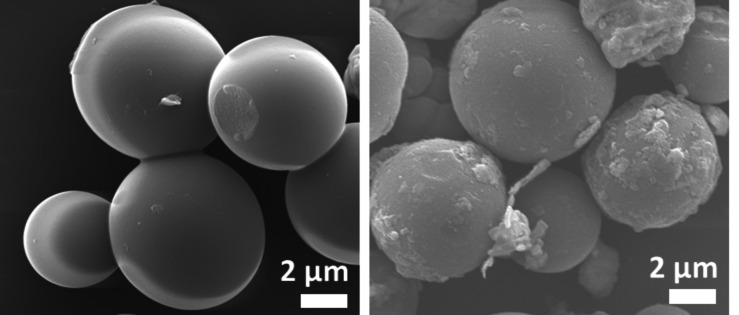
FESEM images of (a) Cu microsphere and (b) Ag‐decorated Cu microsphere.

Figure 2 shows the morphologies of the prepared catalyst that were analyzed using FESEM. The result showed the microsphere of copper ranging from 3 to 6 microns. During deposition of Ag on Cu microspheres, there is no significant change in dimension; however, the surface becomes rough. The EDX mapping confirmed the uniform distribution of Ag nanoparticles over Cu microspheres (Figure S1). The well‐dispersed Ag over the Cu supported the formation of Ag−Cu biphasic boundaries. The percentage of Cu and Ag were found to be 75.4 wt % and 24.6 wt % (Figure S2). Around 100 nm silver nanoparticles were obtained when a solution of Ag^+^ ions was reduced using UV‐light (Figure S3). The newly formed Ag nuclei undergo nucleation and grow to form nanoparticles. HRTEM image shows the deposition of silver over copper microsphere and EDX analysis confirmed the uniform distribution of silver nanoparticles over copper microsphere (Figure S4).

**Figure 3:**
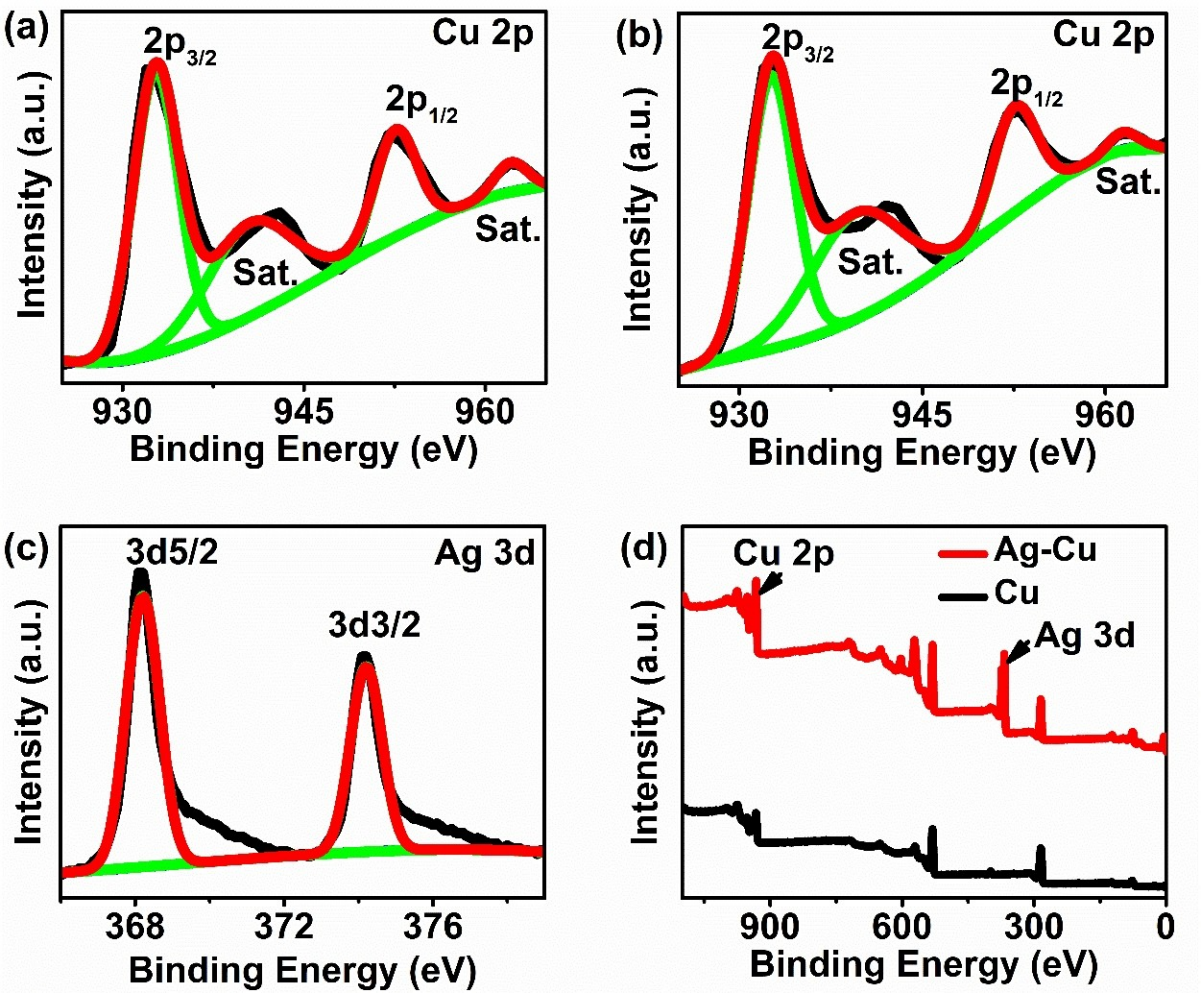
XPS spectra of (a) Cu (2p) for Cu microspheres, (b)‐(c) Cu (2p), and Ag (3d) region for Ag‐decorated Cu microsphere and (d) survey spectra of Cu and Ag‐decorated Cu microspheres.

The composition and the chemical state of elements in the prepared catalyst, was studied using XPS. The peaks at 932.6 eV and 952.5 eV after peak fitting confirm the Cu(0) 2p peaks, and the weak peaks at 939.5 eV and 962.08 eV were attributed to the Cu^2+^ ions peaks (Figure 3a and b). The presence of Cu^2+^ ion peaks is due to the oxidation of the copper surface throughout exposure to air.^[24]^ Similarly, the peaks at 368.21 eV and 374.21 eV are attributed to Ag 3d_5/2_ and Ag 3d_3/2_ peaks of metallic Ag (Figure 3c). There is a slight shifting in binding energy in the case of silver atoms towards lower energy because of electron acceptance compared to the pure Ag (3d) peaks, confirming the electron transfer between Cu and Ag atoms.^[21]^

**Figure 4:**
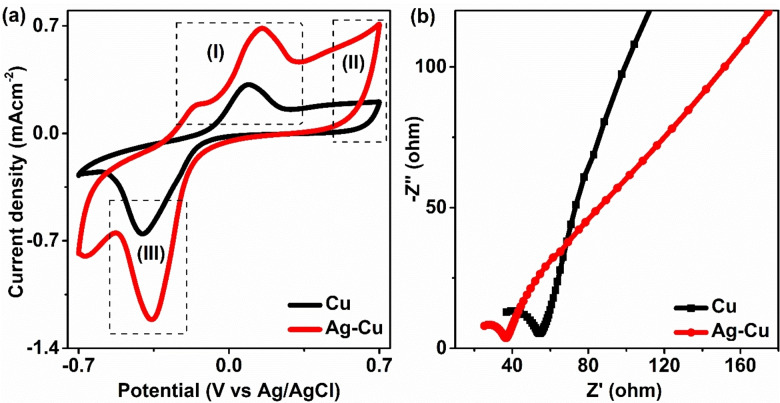
(a) Cyclic Voltammogram for Cu microspheres and Ag decorated Cu microspheres and (b) Electrochemical impedance spectroscopy (EIS) graph of Cu microspheres and Ag decorated Cu microspheres.

Figure 4a shows the cyclic voltammetry curve of the as‐prepared catalyst. The oxidation peak at region (I) is associated with the formation of Cu^+^ ion and Cu^2+^ ion.^[25]^ It shows that Ag is well distributed and does not cover the Cu sphere. The Cu sphere is accessible for the reaction. Similarly, region (II) is associated with the presence of Ag over the surface of Cu, and similar trends were observed at different scan rates (Figure S5). The increase in current while increasing the scan rate is due to an increase in ionic/electronic response. Similarly, region (III) is associated with the reduction peaks of Cu^2+^ to metallic Cu. Shifting and increase in peak position in the reduction region is related to an increase in enrichment of Cu at the surface level.

**Figure 5:**
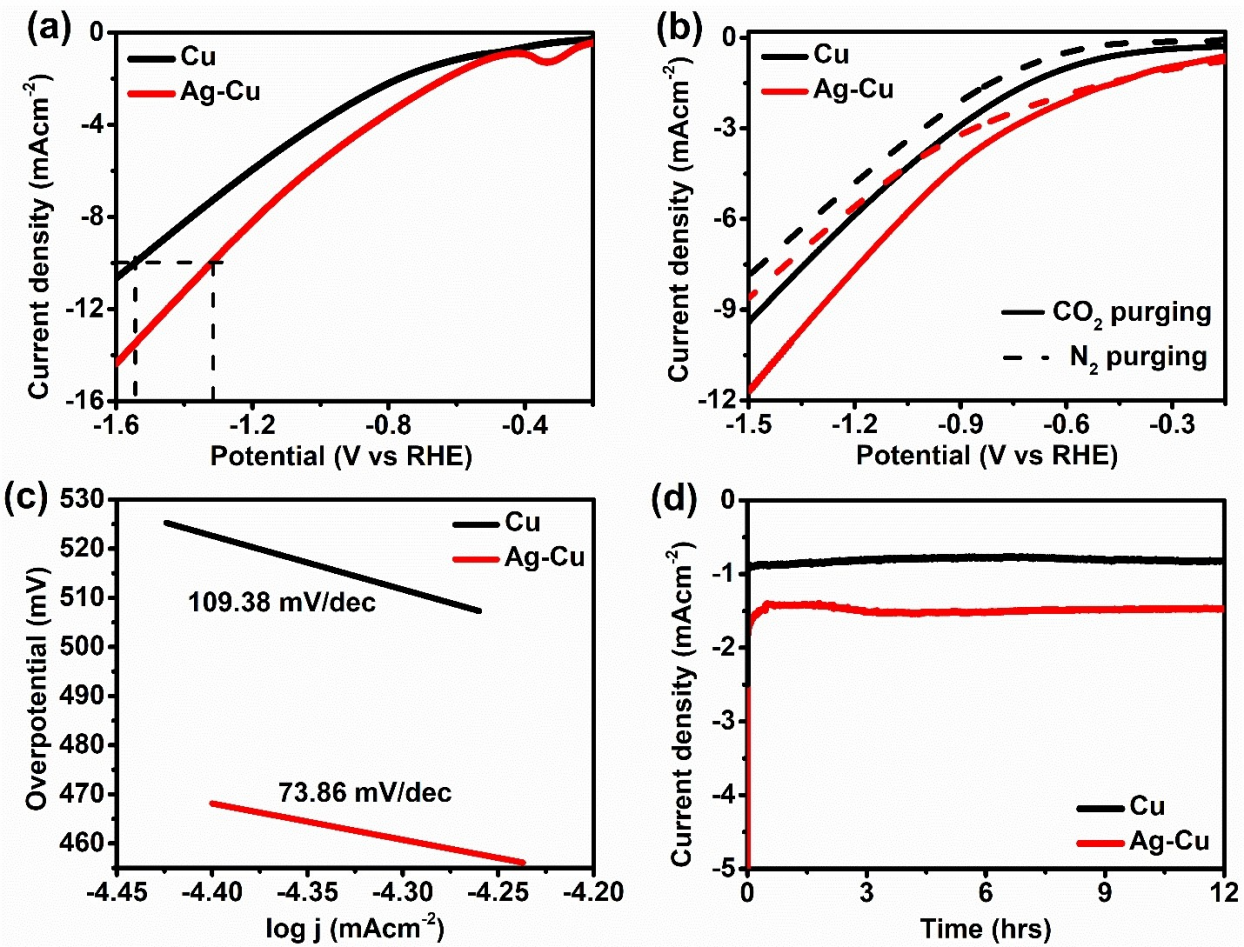
(a) Linear sweep voltammetry (LSV), (b)before purging and after CO_2_ and N_2_ purging, (c) Tafel plot with a linear fit for catalyst and (d) stability of the prepared catalyst.

Electrochemical impedance spectroscopy (EIS) data were used to investigate the catalyst's charge transfer kinetics. The rates of charge transfer and the inherent catalytic activity of the electrocatalysts are directly correlated with the diameter of the semicircle in the high‐frequency region of electrochemical impedance spectroscopy (EIS). The representation of impedance is separated into two parts: the imaginary part (Z′′ or Z_imag_) expressed on the y‐axis and the real part (Z′ or Z_real_) shown on the x‐axis. This creates a Nyquist plot, in which each point plotted on the graph represents an impedance at a particular frequency, with the imaginary part (Z′′) being represented as negative.[^26^, ^27^] The smaller semicircle of the silver‐decorated copper microsphere compared to the copper microsphere was realized as shown in Figure 4b. The charge transfer resistance is represented by the semicircle diameter, the smaller the semicircle, the lower the charge transfer resistance and the higher the electron transfer kinetics during CO_2_ reduction. The equivalent circuit is shown in Figure S6.^[28]^ The parameters of the equivalent circuit, like solution resistance (R1), charge transfer resistance (R2), and electric double‐layer capacitance (Cdl1) that provide the fitted EIS data, are included in Table S1. The result shows the lower charge transfer resistance of Ag‐decorated Cu with a slight increase in electric double‐layer capacitance. This result indicates that the Ag‐decorated Cu catalyst provides better electrocatalytic performance. This finding showed that adding silver enhanced the catalyst's conductivity and active site, which can increase CO_2_RR activity. In linear sweep voltammetry (LSV), the current increases as more negative potential is applied (Figure 5a and b).

Compared to copper, the silver‐decorated copper catalyst exhibits the maximum current density at the same potential. The higher current density results from faster electron transport and increased energy efficiency. At −0.38 V, there is an increase in current due to the reduction of silver over the copper microsphere.^[19]^ Comparably, in the case of CO_2_ saturated electrolyte, when compared to N_2_ saturated electrolyte (Figure 5b), a more significant current density of silver‐decorated copper microsphere was noted. This result shows the dominance of CO_2_RR over HER. This demonstrates that adding silver to the copper microsphere enhanced the electrochemical activity toward CO_2_ reduction. The catalyst surface kinetics for CO_2_RR were also studied using Tafel slopes. In Figure 5c, the Tafel slope of the silver‐decorated copper microsphere was 73.86 mV/dec, which is much lower than the Tafel slope of the copper microsphere (109.38 mV/dec). This result also supports the evidence that adding silver over the copper microsphere enhanced the catalytic properties of CO_2_RR by increasing the active site.

The Faradaic efficiency (FE) of the Cu and Ag‐decorated Cu microsphere was calculated and compared, as shown in Figure S7. The result shows that FE was increased almost two‐fold when silver was decorated over the copper microsphere. The FE of copper and silver‐decorated copper was 44 % and 70.93 %, respectively, when the potential was −0.50 V vs RHE.

Similarly, hydrogen generation also increased due to the presence of Ag in the copper microsphere. Similar trends were observed in different potentials, as shown in Figure S8. The result showed a higher selectivity of methane production when analyzed in GC with no trace of other products. Table 1 compares our work with the previously published research work, confirming that the result is comparable. Similarly, no liquid product was obtained when analyzing the liquid samples in NMR. The above result completely showed the selectivity of the prepared catalyst toward methane production. Although silver has greater chances of forming CO because of the lower adsorption of CO over the silver surface,^[29]^ a higher amount of copper played a crucial role in selectivity towards methane using CO as an intermediate as binding energy between Cu and *CO intermediate promotes the hydrocarbons. Similarly, the higher selectivity towards methane may also be attributed to the Cu (111) crystalline plane that provides greater active sites for CO adsorption.^[30]^ Introducing the silver nanoparticles provides a more active site for CO, enhancing the electrochemical reduction of CO_2_. The possible pathway for methane production is the adsorption of CO_2_ on the Cu (111) plane followed by protonation to generate activated COOH (*COOH) intermediate via a proton‐coupled electron transfer process (PCET). *COOH intermediate further converted to *CO intermediate via loss of water. Due to the redistribution of electrons at Ag and Cu surfaces, electrons are transferred between copper and silver, which is responsible for the increase in binding strength of *CO to the Cu surface. This step is crucial for the selectivity towards methane. Furthermore, the applied potential favors the protonation of adsorbed *CO triggering the formation of *CHO.[^31^, ^32^] *CHO formed undergoes further protonation into *CH_2_O, and *OCH_3_, which is intermediate through the PCET process. Finally, *OCH_3_ leads to the formation of methane.[^7^, ^23^, ^33^] The synergetic effect between silver and copper might play a key role in the stability of *CO, which is later converted into a key *CHO intermediate that enhances the faradaic efficiency of methane formation.[^31^, ^34^] The stability of the catalyst was also tested for 12 hours, which showed the excellent stability of the catalyst for the electrochemical reduction of CO_2_ (Figure 5d). The stability of the catalyst was also further investigated by XRD analysis and FESEM imaging after the electrochemical testing. The XRD results shown in Figure S9 matched with the freshly prepared catalyst and there was no shifting or change in the intensity of peaks. Similarly, the FESEM of the catalyst after 12 hours is also shown in Figure S10. It also supported the idea that the catalyst could be reused. All these characterization techniques support the excellent stability of the catalyst for the electrochemical reduction of CO_2_.

**Table 1:** Comparison of the catalytic performance of synthesized Cu microsphere with other Cu based catalyst.

Catalyst	Electrolyte	Potential (V vs RHE)	FE (%)	Stability	Ref.
La_2_CuO_4_ perovskite oxide	0.1 M KHCO_3_	−1.4 V	56.3	5 hrs	[35]
Cu_68_Ag_32_	0.5 M KHCO_3_	−1.17 V	60	50 hrs	[31]
Nano‐twinned Cu	0.1 M KHCO_3_	−1.22 V	86.1	–	[36]
Organic molecule modified Cu	0.1 M KHCO_3_	−1.1 V	50	1.39 hrs	[37]
Cu_4_‐MFU‐4 l	0.1 M NaHCO_3_	−1.3 V	88	>20 hrs	[38]
Cu‐Co electrode	0.1 M KHCO_3_	−1.19 V	47.7	–	[39]
Au‐Cu	0.1 M KHCO_3_	–	56	–	[40]
Cu_2_O	0.5 M KHCO_3_	−0.3 V	76.61	11 hrs	[41]
Cu‐CeO_2_	0.1 M KHCO_3_	−1.0 V	50	22 hrs	[42]
Metal (Zn, Cu, Ni) Indium Sulfide	0.5 M KHCO_3_	−0.6 V	80.11	20 hrs	[43]
Cu‐CeO_2_‐350	1 M KOH	−0.89 V	∼30	4 hrs	[44]
Cu‐CeO_2_	0.1 M KHCO_3_	−1.8 V	58	2.5 hrs	[45]
Ag‐Cu	0.5 M KHCO_3_	−0.5 V	70	12 hrs	This work

## 3 Conclusions

The photoreduction of silver ions on the preformed microspheres of Cu leads to the formation of Ag‐decorated Cu microspheres to boost the electrochemical property. It showed remarkable selectivity towards the methane with an FE of 70.94 % when compared to the copper microsphere (44 %) because of the stability of *CO intermediate that leads to *CHO, a key intermediate for further reduction leading towards the selectivity of methane production. Moreover, the silver decorated catalyst showed lower overpotential with 10 mA/cm^2^ current density. The enhancement of electrochemical measurements is attributed to the addition of silver over the copper microsphere. The silver‐decorated copper microsphere demonstrated an excellent selectivity for methane through CO_2_RR, which paved the way for tuning the catalyst selectivity for the desired product.

## Conflict of Interests

The authors declare that they have no known competing financial interests or personal relationships that could have appeared to influence the work reported in this paper.

4

## Supporting information

As a service to our authors and readers, this journal provides supporting information supplied by the authors. Such materials are peer reviewed and may be re‐organized for online delivery, but are not copy‐edited or typeset. Technical support issues arising from supporting information (other than missing files) should be addressed to the authors.

Supporting Information

## Data Availability

The data generated or analyzed during this study are available from the corresponding author upon request.
