# Supplementary material for: Selective Electrochemical Conversion of CO2 into Methane on Ag‐Decorated Copper Microsphere
Source: ChemistryOpen. 2024 Oct 24;14(1):e202400173. doi: 10.1002/open.202400173 (PMC11726645; doi:10.1002/open.202400173)
Supplement: Supplementary file 1 — Supporting Information [file OPEN-14-e202400173-s001.pdf]

ChemistryOpen

Supporting Information

Selective Electrochemical Conversion of CO_2 into Methane on Ag-Decorated Copper Microsphere

Rabin Dahal, Rohit Srivastava, and Bishnu Prasad Bastakoti*

Supplementary Information

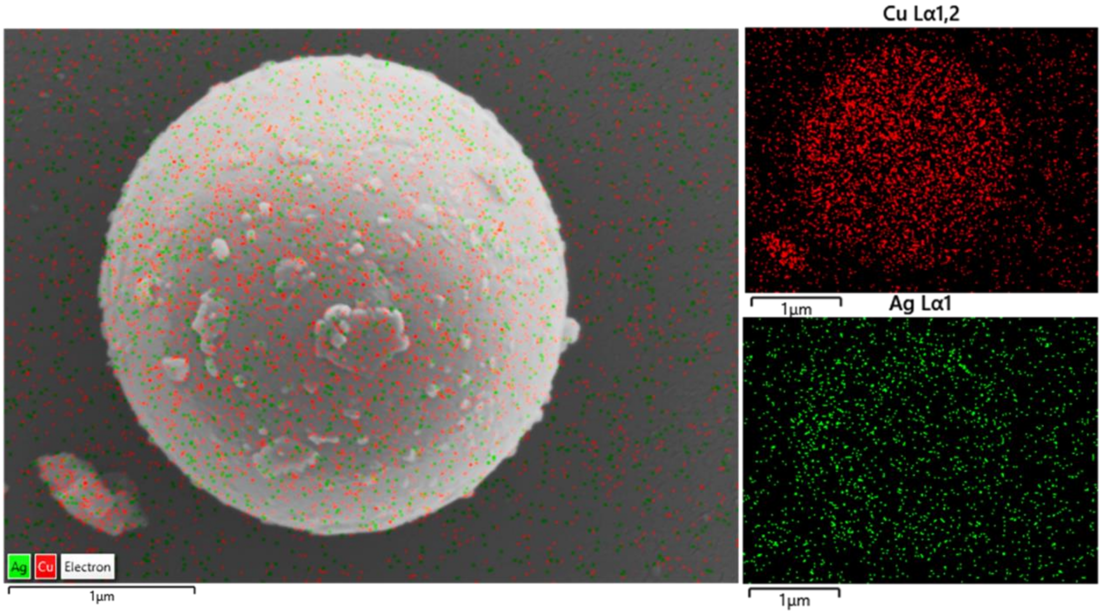

Figure S1. FESEM elemental mapping of Ag-decorated Cu microsphere.

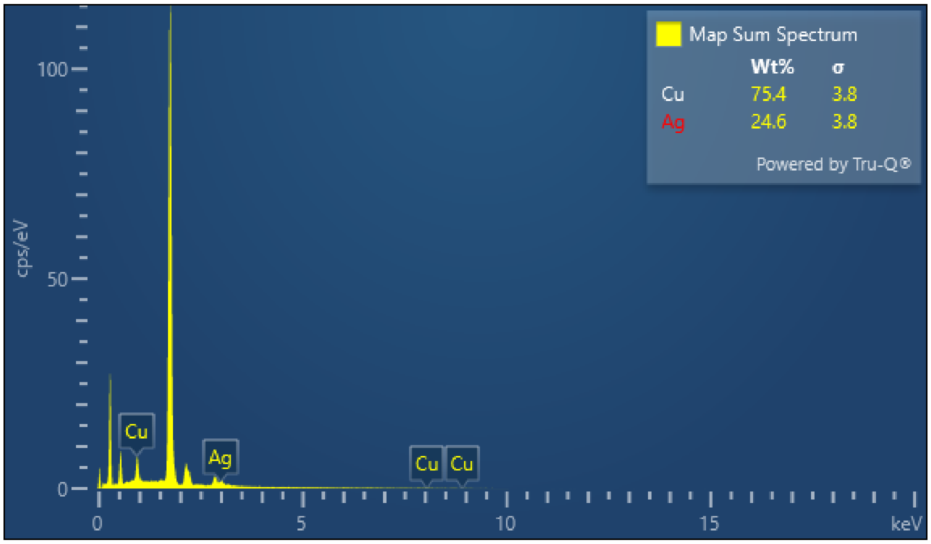

Figure S2: EDX spectrum of Ag-decorated Cu microsphere.

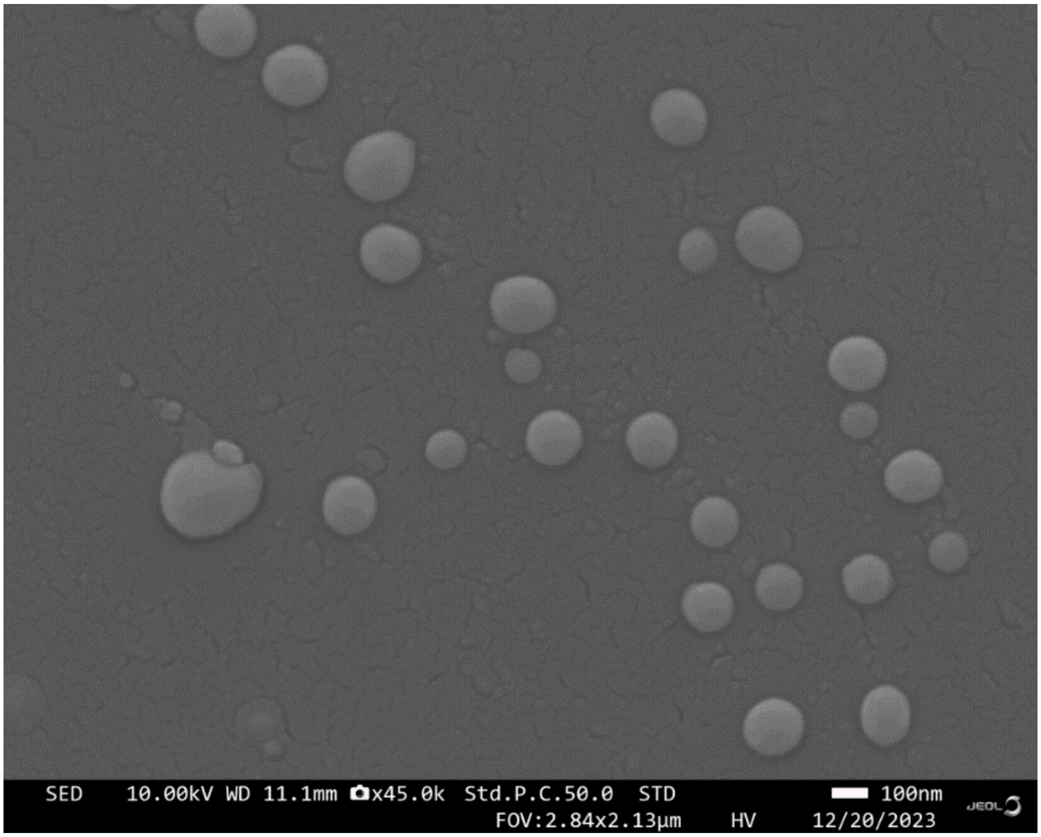

Figure S3. SEM image of Ag NPs synthesized using UV light.

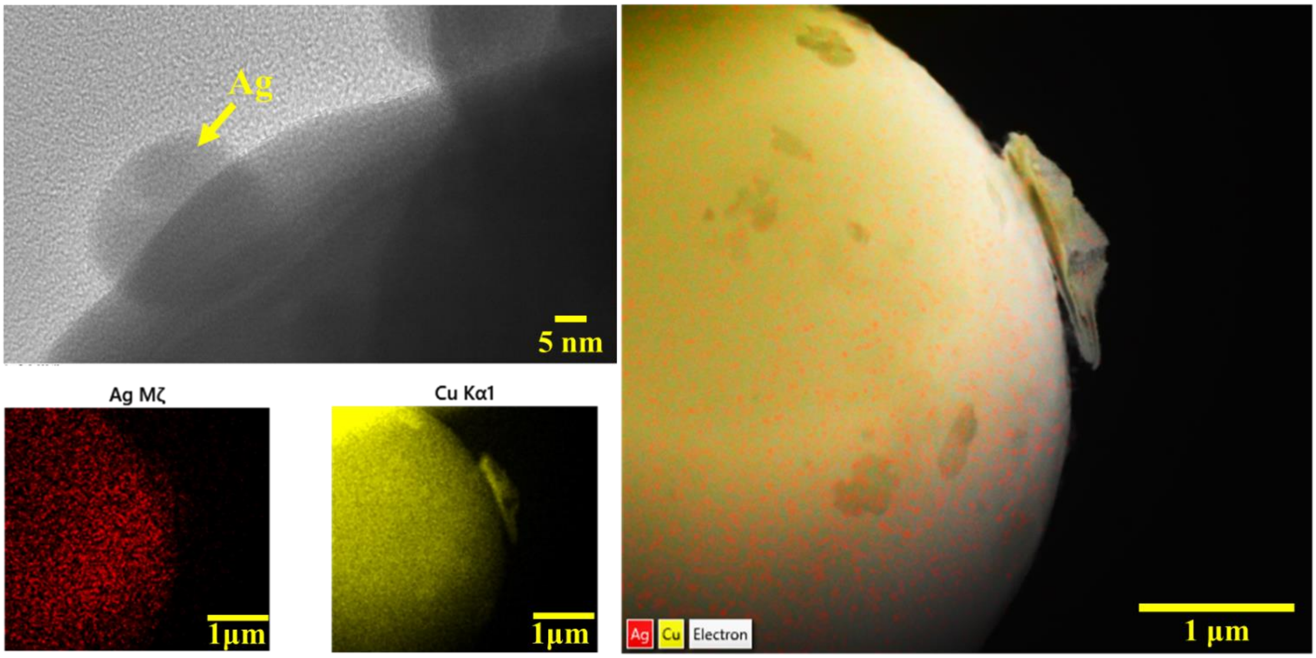

Figure S4. HRTEM image and EDX mapping of Ag-Cu ms.

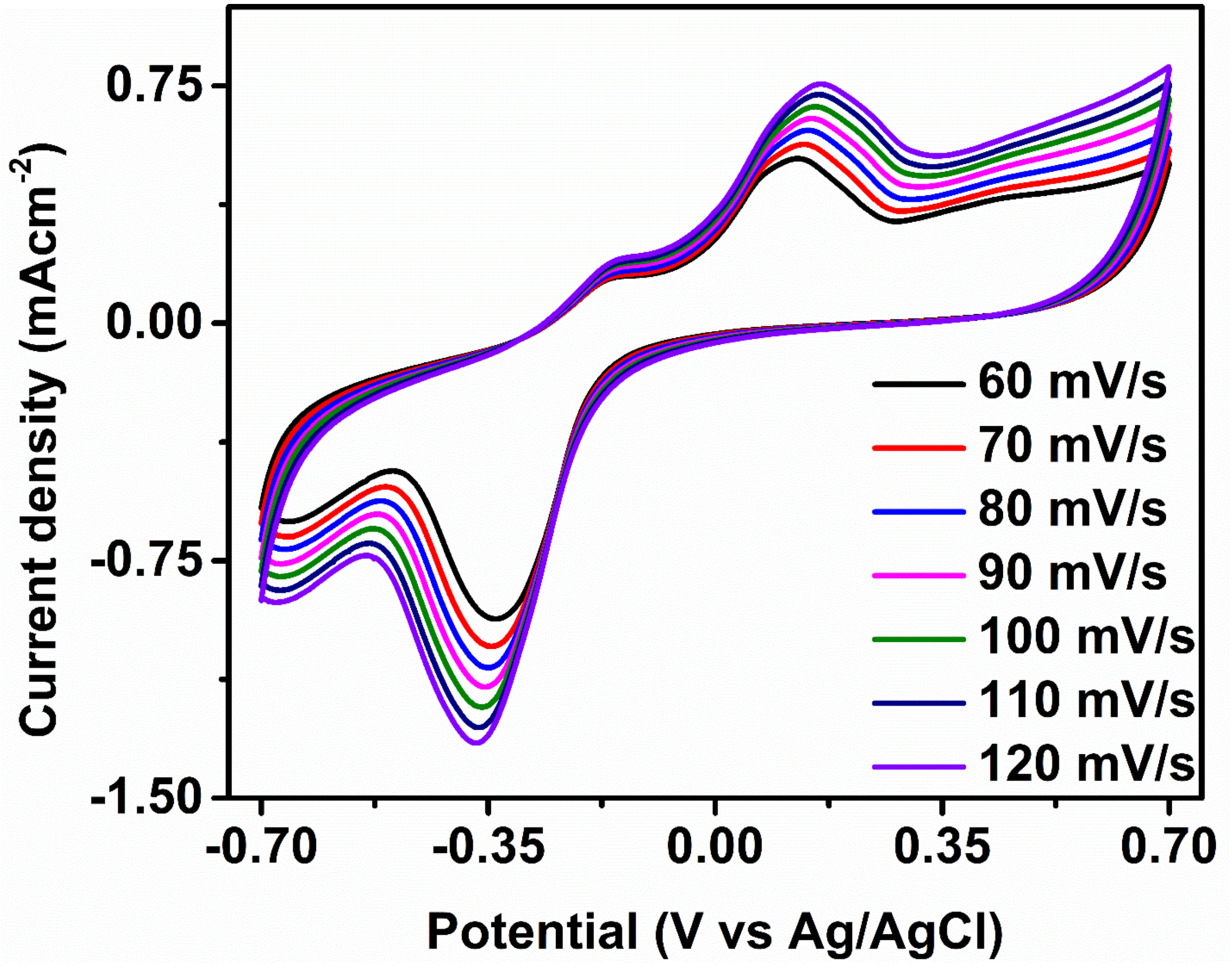

Figure S5. Cyclic voltammetry of Ag-decorated Cu microspheres at different scan rates.

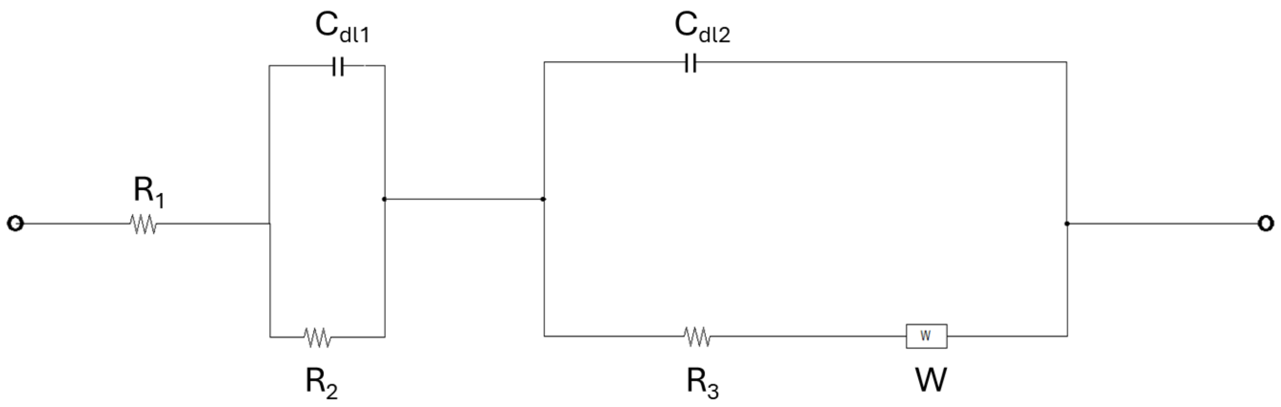

Figure S6. Equivalent circuits used for the fitting process for both Cu and Ag-Cu microsphere.

Table S1. Equivalent series resistance values of samples derived from the EIS analysis.

Electrode	R1 (Ω) (Solution resistance)	R2 (Ω) (Charge transfer resistance)	R3 (Ω) (Lower faradaic resistance)	C_{dl1} (double layer capacitance)	W (σ) (Warburg impedance coefficient)	C_{dl2} (pseudocapacitance)
Cu ms	31.47	26.1	253.4	1.17×10^{-7}	0.000485	2.29×10^{-5}
Ag-Cu ms	20.98	16.46	29.45	1.69×10^{-7}	0.00106	1.96×10^{-5}

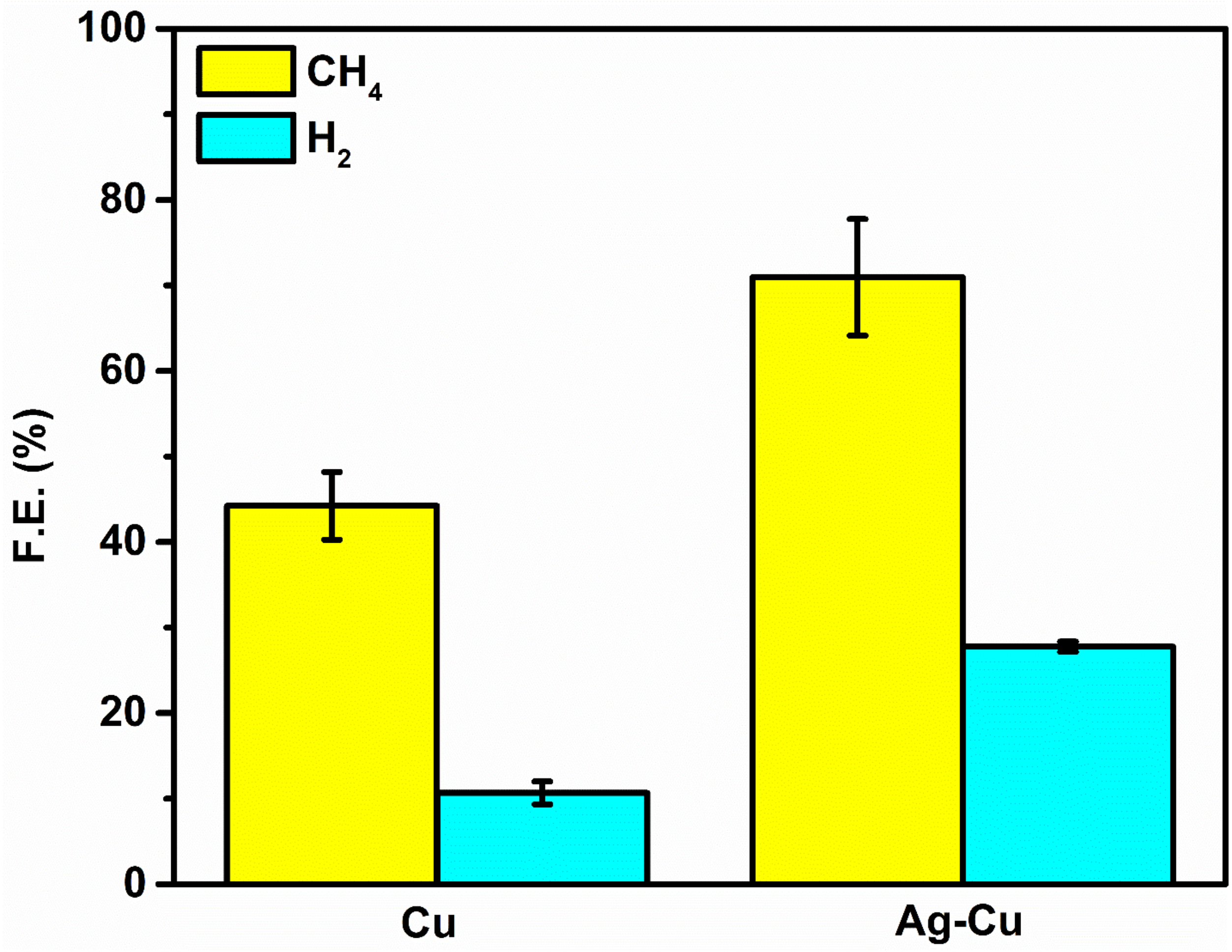

Figure S7. Faradaic efficiency of Cu microspheres and Ag-decorated Cu microspheres for the production of CH_4 at -0.50V vs RHE.

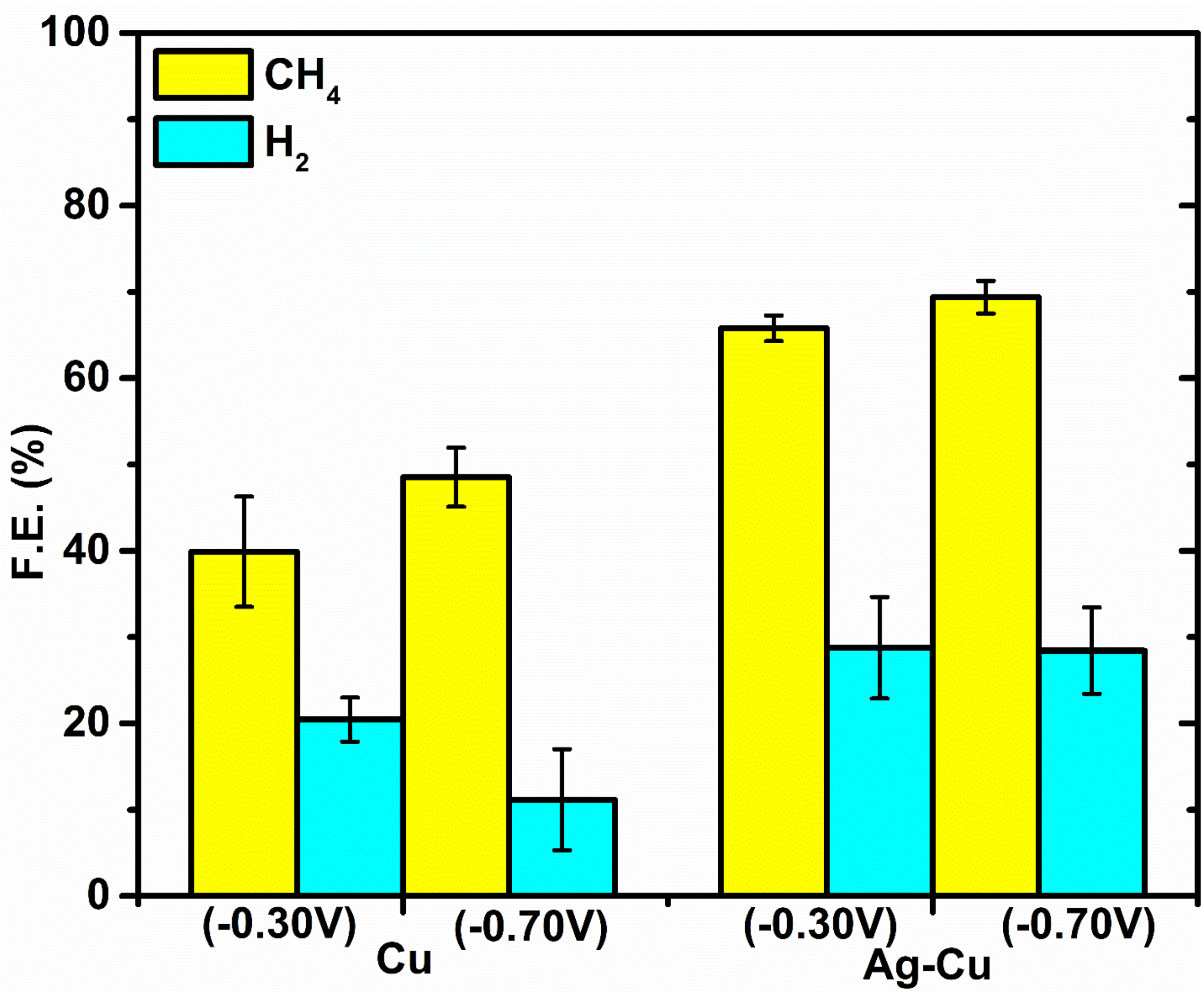

Figure S8. Faradaic efficiency of Cu microspheres and Ag-decorated Cu microspheres for the production of CH_4 at different potentials.

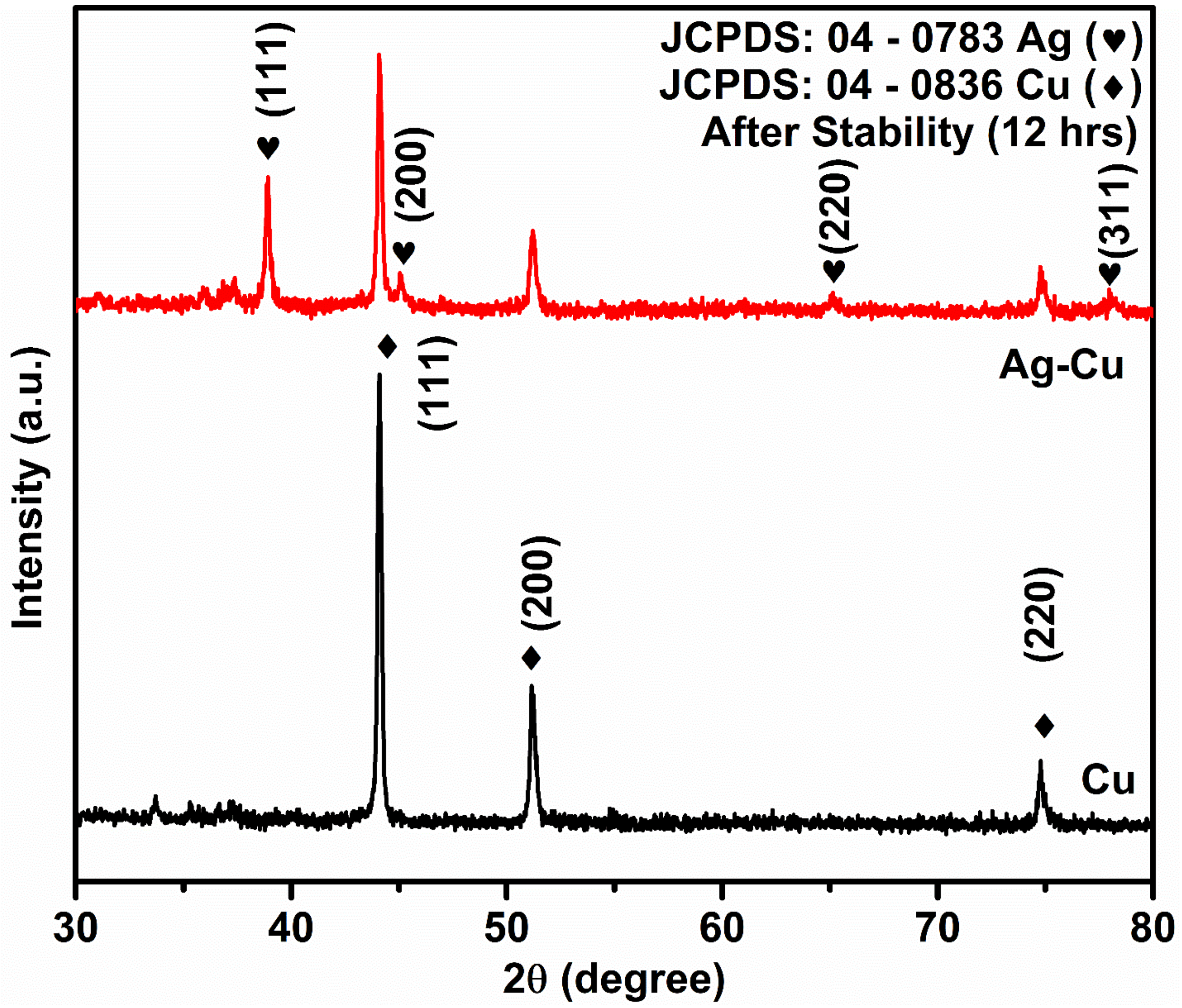

Figure S9: XRD of Cu microspheres and Ag-decorated Cu microspheres after the stability test for 12 hours.

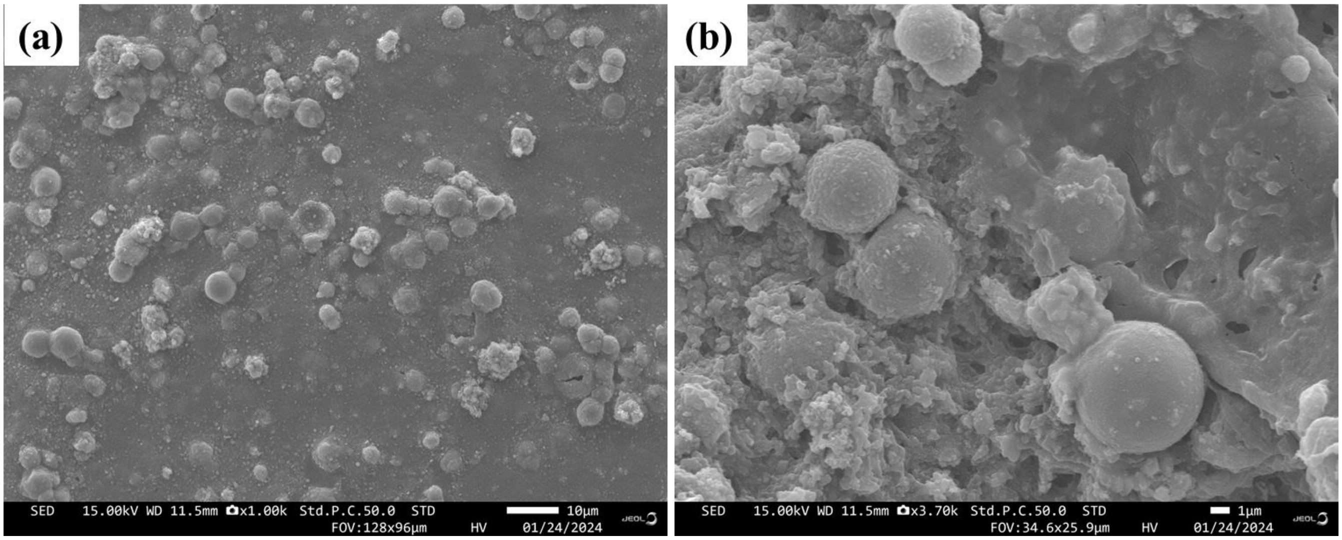

Figure S10: SEM image of (a) Cu microspheres and (b) Ag-decorated Cu microspheres.
